# Use and barriers to the use of telehealth services in the Arab population in Israel: a cross sectional survey

**DOI:** 10.1186/s13584-023-00569-6

**Published:** 2023-05-23

**Authors:** Nadav Penn, Michal Laron

**Affiliations:** Health Policy Team, The Myers-JDC-Brookdale Institute, JDC Hill, POB 3886, 9103702 Jerusalem, Israel

**Keywords:** Telehealth services, Arab population, Accessibility

## Abstract

**Background:**

Studies conducted in Israel and in other countries show that minority populations typically underuse telehealth services notwithstanding the advantages inherent in the use of these services. The goal of this study was to examine telehealth use patterns and the barriers to the use of telehealth services in the Arab population in Israel, which is a culturally and ethnically varied minority population with a unique language and culture.

**Methods:**

A telephone survey was conducted among a representative sample of the adult Arab population in Israel from October 29 to November 4, 2020. Of the randomly sampled 1,192 adult Israeli Arabs 501 participants responded to the entire questionnaire, representing a response rate of 42%.

**Results:**

The study found that the majority of the adult Arab population in Israel faced no technology or Internet accessibility barriers. Thus, the majority of adult Israeli Arabs (87%) use the Internet on a daily basis and have smartphones (96%) and an Internet connection (93%). However, although they have high technology and Internet accessibility, their use of telehealth services is mostly a telephone appointment with a doctor (66%). At the same time, significantly lower use rates were found with regard to advanced telehealth services delivered through the Internet, e.g., consultation with a health care provider by email or chat (34%) or video chat (8%) and ordering of medications (14%). It was found that Arab Christians are more likely to use digital services than Arab Muslims, even when background characteristics are statistically controlled. Lack of awareness was found to be the major barrier to the use of telehealth services, specifically advanced services such as ordering of medications (23%) and video medical consultation (15%). A high rate of women cited the unmet need for the discreet provision of telehealth services as a barrier to their use of the services. It was also found that the majority of the adult Arab population had no objection in principle to the use of email or chat (75%) or video chat (51%) for consultation with a health care provider. It was further found that facilitating factors promoting the use of telehealth services include previous acquaintance with the health care provider, a stable internet infrastructure, the provision of the services in the Arabis language, guidance in the use of the service, a recommendation by a health care provider, and the participation of a family member in the online medical consultation.

**Conclusions:**

The study findings highlight the need for the provision of accessible and customized telehealth services for minority populations. Whether delivered over the phone or through the Internet, the services have to be culturally (for Muslims and Christians) and linguistically (Arabic) adapted, guidance in the use of the services should be provided, and service marketing should be tailored to the target minority population. Specific solutions should be developed for the discreet provision of telehealth services for women, maintaining their privacy in online consultation with a health care provider, while the option of having a family member participate in the online medical consultation should be clearly indicated. In addition, awareness of telehealth services should be enhanced through promotional activities culturally adapted to the Arab society, for instance, through recommendation by the family doctor.

## Background

In recent years and especially since the onset of the COVID-19 pandemic, health care systems in Israel and worldwide have been acting to promote the use of telehealth services by patients and health care providers [1, 2]. The wide-ranging efforts for the incorporation of telehealth services are driven by the research-backed belief that telehealth services can significantly improve the quality and accessibility of health care [3]. Unlike the traditional in-person health care services, telehealth services allow for increased accessibility and frequency of medical consultation and care while reducing the time and financial resources required for the provision and consumption of the services [4]. Hence, telehealth services can not only improve health care quality but also accommodate large populations that have difficulty accessing in-person health care services, such as communities in remote areas, military personnel, inmates, and people with disabilities [3, 4].

Yet, notwithstanding the potential value of telehealth services for both health plan (HMO—Health Maintenance Organization) members and the health care system, in general, certain population groups typically underuse these services [5–8]. Ethnic minorities, socioeconomically vulnerable and educationally disadvantaged populations (the poor and those without higher education), older adults, and people living alone are less likely to use telehealth services, compared with the general population [5–8]. No clear-cut effect of gender on the consumption of telehealth services has been found in the research published to date. Some studies found no difference between men and women, other studies showed that women consumed more telehealth services while some other studies found that certain telehealth services were consumed by men more than by women [7].

Differences in the use of telehealth services between the Jewish and Arab populations in Israel were manifest even before the COVID-19 pandemic. Telehealth use rates were lower in the Arab population, compared with the Jewish population [9, 10]. In a survey conducted in 2016, 22% of the Jewish respondents reported consulting online with a family doctor in the previous two years, compared with 13% of the Arab respondents [9]. A follow-up survey conducted in 2018 found that 41% of the Jewish respondents contacted a doctor, whether a family doctor or a medical specialist, for online consultation in the previous two years, compared with 17% of the Arab respondents [11]. Hence, in the two-year period between the two surveys, telehealth use rates in the Jewish population increased by 86%, compared with 31% in the Arab population. That is, the differences in telehealth use rates between the Jewish and Arab populations apparently increased over that period. However, this apparent increase may be attributed in part to the phrasing of the survey questions—with reference to the family doctor, in the first survey, and with reference to any doctor, in the second survey, combined with the fact that use rate of specialists is higher among Jews than among Arabs. The apparent increase in the difference between the two populations may thus have been not as marked as noted above.

Differences in the use of telehealth services were also found among Israeli Arabs. A survey conducted in Israel in 2020 found that Jews were four times more likely to use telehealth services than Arab Christians and 11 times more likely than Arab Muslims [10]. The limited use of telehealth services in the Arab population in Israel, compared with the Jewish population, cannot be accounted for by differences in health needs. In fact, the health status of the Arab population in Israel is not as good as that of the Jewish population. Furthermore, given the younger age of the Arab population, compared with the Jewish population, and the high level of digital literacy characteristic of the younger age group, a higher telehealth use rate could have been expected in the Arab population.

The research literature shows that there are various causes for the non-use or under-use of telehealth services, some of which are related to personal preferences, others having to do with barriers in the health care system while other causes are associated with the specific barriers facing health care providers and patients. Among these causes are the absence of adequate infrastructures; the lack of cultural adaptation of the services; personal preferences, views, and attitudes of health care providers and patients; a low level of digital literacy; and a low level of digital health (eHealth) literacy [12, 13]. In most cases, socioeconomically vulnerable population groups are primarily affected, so that differences in the use of telehealth services may not necessarily be ascribed to differences in health needs or personal preferences. In fact, the differences in the use of telehealth services, reflected in under-use by socioeconomically vulnerable population groups, undermine the equity and efficiency of the health care system. Accordingly, health care systems across the world have been acting to reduce or even eliminate the barriers to the use of telehealth services by the general population and, in particular, by specific vulnerable population groups [14].

The aim of the study presented in this paper was to examine telehealth use patterns, the prevalence of the use of telehealth services, and the characteristics of telehealth users in the Arab population in Israel as well as the barriers to the use of telehealth services and the factors facilitating telehealth use in the Arab population in Israel. The findings of the present study are intended to enable policy makers in the health care system and health organizations to identify and characterize the barriers to the use of telehealth services in minority population groups and to assist them in the formulation of policy aimed at reducing gaps in the use of telehealth services between minority groups and the general population and enhancing the accessibility and equity of health care services. As noted, the study findings are related to the Arab population in Israel; however, many of them are probably also relevant to other Western countries where minority population groups have been found to underuse health services, in general, and telehealth services, in particular.

## Methods

### Theoretical model for the examination of barriers to the use of telehealth services

The present study is based on the theoretical model developed by Levin-Zamir and Bertschi [15] to examine media health literacy, with adaptations required for the examination of the barriers to the use of telehealth services. The theoretical model used in the present study addresses three aspects related to the individual—the personal, the circumstantial, and the environmental. The personal aspect of the model concerns socio-demographic characteristics; the circumstantial aspect has to do with the health status and the level of eHealth literacy; and the environmental aspect involves the media environment and the social environment. The media environment refers to the accessibility to the technology required for the implementation of telehealth services, the usability of the services, and their cultural adaptation to the target population. The social environment refers to the social support for the use of telehealth services (including the implementation of health recommendations) and the availability of formal and informal guidance and support frameworks for the use of telehealth services. This paper focuses on the findings related to the environmental aspect.

The study examined specific elements of the environmental aspect, as presented in the model developed by Levin-Zamir and Bertschi [15]. In the context of the media environment, the study examined the ownership of end devices, e.g., smartphone and computer, Internet accessibility, and aspects of cultural adaptation to the target population, e.g., the accessibility of services in the Arabic language, and the option of having a family member present in the online medical consultation while allowing for consultation in privacy, as required. In the context of the social environment, the study examined the availability of supportive frameworks for the implementation and use of telehealth services.

This paper presents study findings regarding telehealth use patterns and the barriers to the use of telehealth services in a minority population (Arabs) in an OECD (Organization for Economic Co-operation and Development) country (Israel) with a universal health care system and advanced digital health services. The native language spoken by the Arab population in Israel (Arabic) is different from the majority language (Hebrew), the education level of the Arab population is lower than that of the general population in Israel, and poverty rates in the Arab population are higher. Also, compared with the general population in Israel, telehealth use rates were found to be lower among Israeli Arabs before the COVID-19 pandemic, [16–18] although they faced no Internet accessibility barriers and were routinely using the Internet [19].

The COVID-19 pandemic has raised global awareness of the need for the development of a technological, technical, legal, and normative infrastructure for the provision of health care services under circumstances that do not allow for in-person health care. The need for social distancing due to the COVID-19 pandemic was a significant catalyst for the increased use of telehealth services, which eliminated the need for direct contact in physical space [20]. In the research context, it presented an opportunity for a thorough examination of the barriers to the use of telehealth services as higher proportions of the population were using telehealth services, whether voluntarily or under constraint, or were advised to do so and thus, were in a position to form an opinion one way or another.

### Telehealth services: definition

Telehealth services are part of the comprehensive field of digital health or eHealth [21]. The broad concept of eHealth encompasses a variety of health-related services delivered by information and communications technologies, including information systems of health care systems, communication channels among physicians enabling consultation and continuity of health care, and information security in health care systems. These services are designed to serve the various actors in the health care system—care providing organizations, individual health care providers, patients, and the Ministry of Health—and are delivered by health-related service providers, health care providers, and the Ministry of Health. In line with the definition suggested by Van Dyk [21], the term telehealth is used in the present study in the meaning of a basket of remotely provided therapeutic, preventive, and health promoting services.

### Data collection

A telephone survey was conducted from October 29 to November 4, 2020, among a representative sample of 501 respondents from the adult Arab population in Israel. The surveyed population was randomly sampled, and quota sampling was applied to ensure adequate representation of the 18 + age group in the Arab population in Israel (excluding the East Jerusalem Arabs and Negev Bedouins residing in unrecognized villages) according to four variables: age, district of residence, religion, and gender. The telephone interviews were conducted by a company specializing in conducting telephone surveys among the Arab population.

The survey questionnaire was pre-tested on 22 respondents and accordingly fine-tuned prior to the main data collection effort. The 22 pre-tested respondents were excluded from the study. Altogether, 1,192 adult Israeli Arabs were sampled, and 501 participants responded to the entire questionnaire, representing a response rate of 42%. 168 additional respondents answered part of the questionnaire and were not included in the study. Compared to the sample population, amongst the respondents who answered part of the questionnaire there was an over representation of residents of the north (70% vs 61%), older adults (65 +) (19% vs 13%), male adults (57% vs 52%), and under-representation of adults 30–49 age group (40% vs 47%).

### Weights of survey data

Although quota sampling was performed, the study population was still unequally represented in the sample following the survey, as reflected, in particular, in the under-representation of Muslim women and men in the 20–29 age group and Druze women in the 20–29 age group.^1^ Sampling weights were thus assigned according to a matrix of four variables: age, district of residence, religion, and gender, where each matrix square represented a unique group in the Arab population with specific characteristics of age, district of residence, religion, and gender. The weight assigned to each group was the ratio between the representation of each group in the Arab population and its representation in the survey sample.

### Ethics

The study was approved by the Ethics Committee of the Myers-JDC-Brookdale Institute. Informed consent was obtained from all participants, by heart, before starting the interview.

## Results

The demographic details of the study population are shown in Table 1. After assigning sampling weights, half of the participants were females, 71% were under the age of 50, 61% were from the North region, and 81% of the respondence were Muslims.

**Table 1:** Distribution of demographic characteristics (after weighting) (%) n = 501

Variable	Description	%
Age		
	18–29	31
	30–49	40
	50–64	19
	65 +	10
Gender	Male	50
	Female	50
Region	North	61
	Haifa	12
	Center, Jerusalem, and Tel Aviv	14
	South	13
Religion	Muslims	81
	Christians	9
	Druze	10

The distribution of the sample according to membership in a health plan is similar to the distribution of residents of Arab urban settlements in Israel. Nevertheless, in the sample there is an Overrepresentation for academics (28% vs. 19%), those with high incomes (over 14,000 NIS 25% vs. 13%), and those reporting on good health (90% vs. 78%).^2^

### Internet accessibility and telehealth use patterns

Internet accessibility is a prerequisite for the consumption of telehealth services. Specifically, access to an end device, e.g., a smartphone, and Internet connection are required as well as Internet literacy, i.e., proficiency in the use of the Internet for information seeking and communication as well as for various routine tasks (bill paying, filling out forms, etc.).

**Fig. 1:**
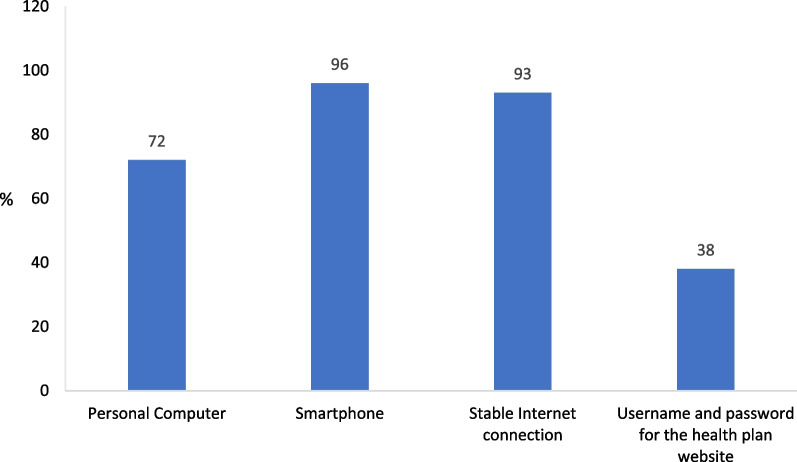
Ownership of Internet access devices (%) n = 501

As shown in Fig. 1, 96% of adult Israeli Arabs have smartphones and 93% have a stable Internet connection. Also, as shown in Table 2, 87% use the Internet on a daily basis while 6% never use the Internet.

**Table 2:** Frequency of Internet use (%) n = 501

Frequency of use	%
On a daily basis	87.0
Several times a week	5.5
Once a week	1.0
Never	6.0

**Fig. 2:**
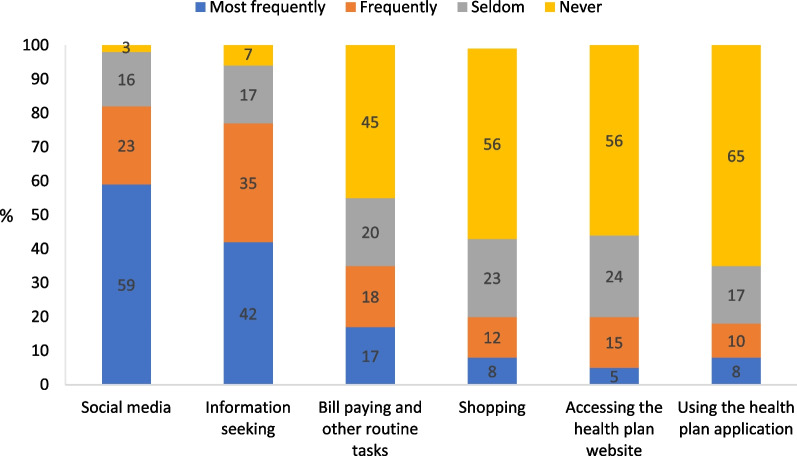
Frequency of Internet use classified by use type (%) n = 470

Surfing the social media is the most popular Internet use in the Arab population in Israel: 59% of the respondents reported a frequent use of the social media while only 3% reported that they never used the social media (Fig. 2). At the same time, the use of the Internet for performing routine tasks (e.g., bill paying, commodity shopping, etc.) or for obtaining medical information from the health plan is rather limited. Thus, 45% of the respondents reported that they never used the Internet to pay bills or for other routine tasks; 56% never accessed a health plan website; and 65% never used a health plan application. Only 38% of the respondents have a username and password for a health plan website, a percentage that is compatible with the low use rates of the health plan website and application (Fig. 1). While the online services of the health plans can be accessed even without a username and password—by a one-time password, on the basis of an ID number or some other identifier—a username and password facilitate the consumption of the services.

**Fig. 3:**
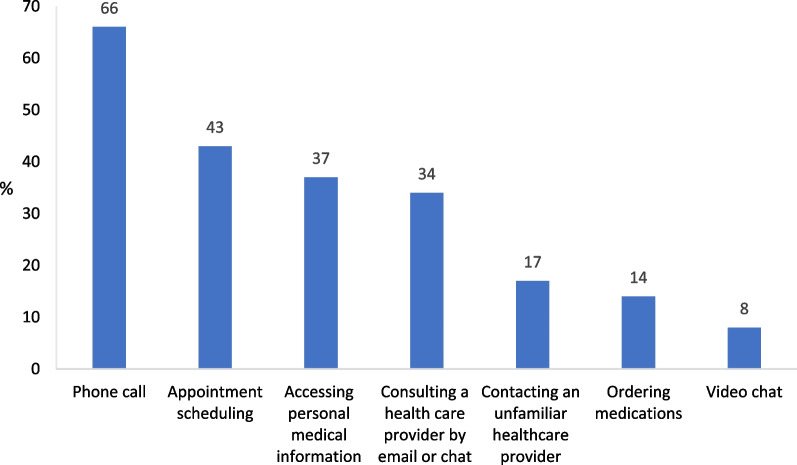
Telehealth use rates in the previous year (%) n = 501. The percentage of respondents who used the specific type of service at least once

The smartphone is the most popular device used for the consumption of telehealth services: 66% of the respondents reported consulting with a familiar health care provider over the phone in the previous year (Fig. 3); online services for appointment scheduling, accessing personal medical information, and consulting a familiar health care provider by email or chat were used by 43%, 37%, and 34% of the respondents, respectively. At the same time, other online services, e.g., consulting a familiar health care provider by video chat, ordering medications, and contacting an unfamiliar health care provider (whether over the phone, by email or chat, or by video chat) were used by 8%, 14%, and 17% of the respondents, respectively—the lowest use rates among the services examined.

### Barriers

In addition to the questions on telehealth use patterns in the previous year, two hypothetic scenarios were presented to the respondents in order to examine their attitudes about the use of telehealth services and their future willingness to use telehealth services: 1) consultation with a familiar health care provider by email or chat; 2) consultation with an unfamiliar health care provider by video chat. In each case, the respondents were asked to envisage and respond to a situation where they are in need of a certain medical service and are offered a telehealth service.

**Fig. 4:**
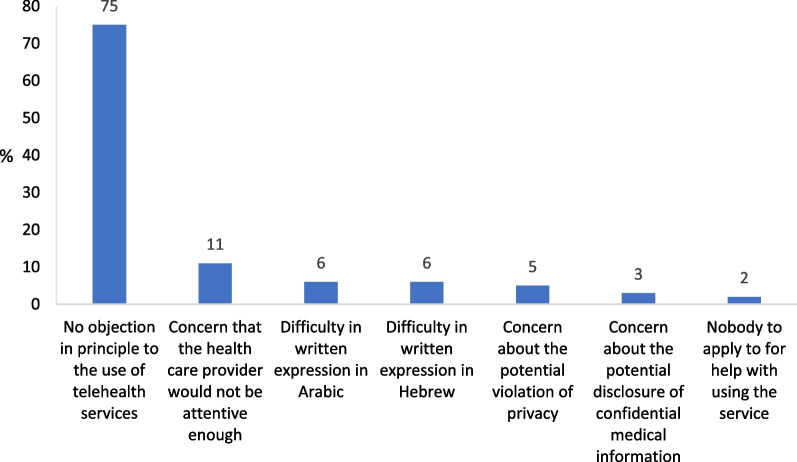
Barriers to consultation with the family doctor by email or chat (%) n = 501 (multiple selection)

**Fig. 5:**
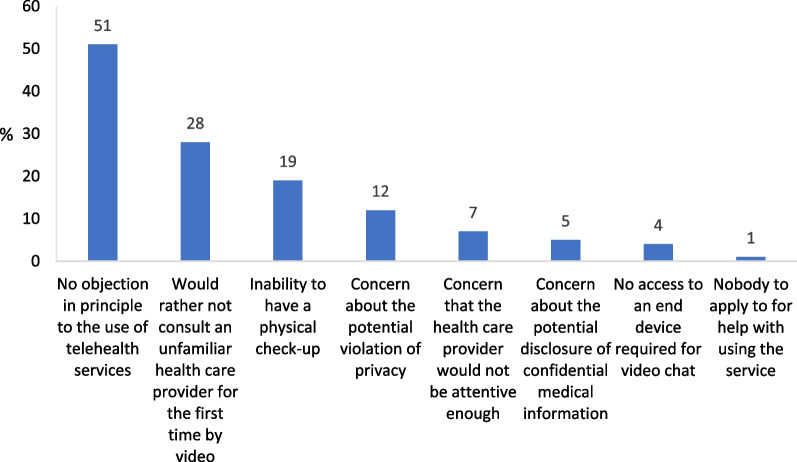
Barriers to consultation with an unfamiliar medical specialist by video chat (%) n = 501 (multiple selection)

With reference to both cases, the majority of respondents said that they had no objection in principle to the use of telehealth services, whether for consulting a familiar health care provider by email or chat (75%) or for consulting an unfamiliar health care provider by video chat (51%) (Figs. 4 and 5, respectively).

As for the option of consultation with a familiar health care provider by email or chat, the main barriers cited by the respondents were: concern that the health care provider would not be attentive enough (11%); difficulty in written expression in Arabic (6%) or in Hebrew (6%); and concern about the potential violation of privacy (5%) (Fig. 4). Analysis of the barriers by income shows that the barrier of difficulty in written expression (in Arabic or Hebrew) is manifested most significantly in low-income households (with a monthly income of up to 4,500 NIS—the low income decile): 26% of the low-income respondents cited the difficulty in written expression in Arabic as a barrier to consultation with a health care provider by email or chat (p = 0.001) while 18% cited the difficulty in written expression in Hebrew as a barrier to consultation by email or chat (p = 0.001).

As for consultation with an unfamiliar health care provider by video chat, 28% said that they would rather not consult an unfamiliar health care provider for the first time by video. Other major barriers cited were: the inability to have a physical check-up (19%); concern about the potential violation of privacy (12%); and concern that the health care provider would not be attentive enough (7%) (Fig. 5). It is worth noting that the majority of respondents were not concerned about the potential disclosure of confidential medical information and that only few said that they had nobody to apply to for help with using the service (Fig. 4, 5). Analysis by gender of the findings related to the barrier of potential violation of privacy shows that women are more concerned about it than men (p = 0.006). While 8% of the men are concerned about the potential violation of privacy in an online consultation with an unfamiliar health care provider, almost double the percentage of women (15%) are concerned about the potential violation of privacy.

### Barriers to the use of telehealth services and facilitating factors

Another way to identify the barriers to the use of telehealth services is to examine the facilitating factors promoting or likely to promote the use of the services. Thus, by adopting a positive approach and asking respondents about facilitating factors rather than about barriers to the use of telehealth services, specifically barriers under their control, they are encouraged to share their use experience and attitudes more freely. This positive approach is generally effective and, in particular, it is effective in the Arab society.^3^

**Fig. 6:**
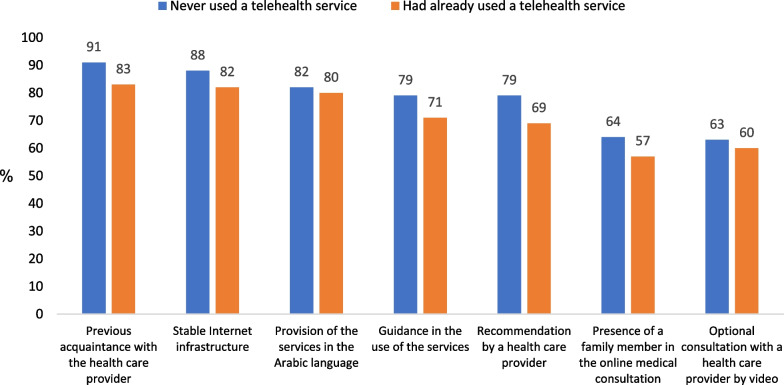
Factors facilitating the use of telehealth services (%) n = 501 (multiple selection)

As shown in Fig. 6, the majority of respondents noted that most of the factors examined facilitated or were likely to facilitate their use of telehealth services. The factors perceived as particularly facilitating were previous acquaintance with the health care provider, a stable Internet infrastructure, and provision of the services in the Arabic language. Higher percentages of those who had never used the services believed that the examined factors would facilitate their use of the services, compared with those who had already used the services. At the same time, both groups, the non-users and the users, rated the facilitating factors in a similar way. The most significant rating differences between the groups were found with regard to the following factors: a recommendation by a health care provider (10% more of the non-users cited it as a facilitating factor); guidance in the use of the services (8% more); the presence of a family member in the online medical consultation (7% more); and previous acquaintance with the health care provider (8% more).

## Discussion

Telehealth services can potentially empower patients [3, 4]. Telehealth services increase accessibility to general and personal medical information, facilitate consultation with the family doctor or with medical specialists who cannot be visited in person, enable guidance on health issues (e.g., child development), spare the need for visiting a clinic or a pharmacy, and minimize waiting times [3, 4]. In the case of a pandemic, telehealth services are helpful in protecting public health and reducing the prospects of infection and spread of diseases [22]. Hence, a low level of accessibility or under-use of telehealth services by certain population groups could further increase health gaps in the population [23].

The present study examined telehealth use patterns and barriers to the use of telehealth services in the Arab population in Israel, a minority population characterized by under-use of health services, in general, and telehealth services, in particular [24, 25]. The study is based on the theoretical model developed by Levin-Zamir and Bertschi [15] and specifically on the environmental aspect of the model, which is at the focus of this paper. The environmental aspect involves the media environment and the social environment. As noted above, the media environment refers to the accessibility to the required technology, the usability of the services, and their cultural adaptation to the target population. It has been long established that accessibility deployment of health services is a key element in increasing health service utilization [26]. As noted above it was also found that a lack of media accessibility could increase health gaps in certain population groups [23].

Furthermore, a 2018 research paper found that fewer Arabs in Israel tend to use the internet for procedural/functional purposes (e.g., filling out forms and paying online), compared to Israeli-Jews [19].

The study found that the media environment of the Arab population in Israel is only in part conducive to the use of telehealth services. While the majority of Israeli Arabs have accessibility to the required technology, which is a prerequisite for the consumption of telehealth services, with 96% of adult Israeli Arabs in possession of a smartphone and 93% enjoying a stable Internet connection, the findings indicate that cultural adaptation, specifically the adaptation of the services to the local Muslim population, is inadequate. Thus, it was found that Arab Christians are more likely to use digital services for various routine tasks than Arab Muslims—as shown even when background characteristics, e.g., education, are statistically controlled. Given the cultural differences between the two groups [27], and the known effect of the social and cultural environment on the usage of health services [28–30], cultural adaptation to the Muslim Arab population in Israel is specifically required to ensure its accessibility to advanced telehealth services. In the broader context, the findings indicate the importance of considering the unique characteristics and needs of specific groups in minority populations, in Israel as well as in other countries.

Another key finding indicating the importance of cultural adaptation to the target population is the high rate of women who cited the unmet need for the discreet provision of telehealth services as a barrier to their use of the services. This is even more important considering the fact that more women in Israel inclined to waive a health service, compared to men [31]. Specific solutions should thus be developed to enable women to consult online with a health care provider while maintaining their privacy.

These findings regarding the cultural and linguistic accessibility gaps in the health care system in Israel, which appears to be discriminatory in relation to the Arab population, are compatible with research findings reported in the literature. Chernichovsky et al. [32] found that Muslims, Christians, and Druze in Israel have difficulty to access information on health care rights and benefits, more so than Jews. Furthermore, compared with Israeli Jews, a higher percentage of Israeli Arabs—Muslims, Christians, and Druze—report difficulties in accessing health care services due to language barriers [32].

Another aspect of the media environment is the usability of the services, i.e., the service accessibility and ease of use—factors that are relevant to the general population and, in particular, to minority population groups with a unique language and culture [33]. As shown in this study, about two thirds of the respondents contacted a health care provider by phone while lower use rates were found with regard to advanced telehealth services, e.g., consultation with a health care provider by video chat and the ordering of medications. A linguistically adapted, user-friendly service is thus more likely to be commonly used [34]. The low use rates of advanced telehealth services in the Arab population in Israel may be attributed, at least in part, to the fact that the online services offered by the health plans do not support data entry in Arabic. A high percentage of the respondents cited the provision of services in Arabic as a facilitating factor that could promote service use while only few explicitly noted that language was a barrier to the use of the services. In any case, the language barrier was found to affect a higher percentage of the low-income Arab population.

As for the social environment, as noted, it refers to the importance of social support for the use of telehealth services (including the implementation of health recommendations) and the availability of formal and informal guidance and support frameworks for the use of telehealth services [15, 35]. As shown in this study, lack of awareness is a major barrier to the use of telehealth services in the Arab population in Israel. Thus, a high percentage of the respondents were unaware of some of the available telehealth services, specifically recently introduced services such as the online ordering of medications—a service with which 23% of the respondents were unfamiliar. A higher percentage of the respondents noted that a recommendation by a health care provider, guidance in the use of the services, and previous acquaintance with the health care provider were facilitating factors likely to promote or already promoting their use of the services. Given the low telehealth awareness rates and the importance of a recommendation by the health care provider, service marketing should be culturally adapted to the Arab society, for example, by assigning to the family doctor the task of direct marketing of the services.

In the context of the social environment, the importance of support for the use of the services, whether by family members or by the health care provider, should also be noted [28, 30]. More than half of the respondents said that the presence of a family member in the online medical consultation was likely to promote or already promoting their use of the service. Hence, as part of the required cultural adaptation and so as to enhance the use of telehealth services in the Arab population in Israel, the option of having a family member present in the online medical consultation should be offered and clearly indicated.

The present study is the first thorough examination of telehealth use patterns in the Arab population in Israel. Further research using qualitative tools such as focus groups and in-depth interviews with patients and health care providers would complement the picture and enable better understanding of the cultural barriers to the incorporation of telehealth services in the Arab population in Israel.

### Study limitations

The study sample is biased by the over-representation of population segments with higher education and high-income groups, where the use of telehealth services is more common. A more adequate representation of these groups in the sample in proportion to their share in the population would most probably yield lower telehealth use rates and allow for the identification of more barriers to the use of the services than found in the present study. Furthermore, as noted above, the East Jerusalem Arabs and Negev Bedouins residing in unrecognized villages—two population groups more likely to be affected by cultural and specifically language barriers—were excluded from the study population and should be included in future research.

## Conclusions

In view of the study findings, several recommendations are suggested. While the recommendations detailed below are all relevant to the promotion of telehealth use in the general population, the first three are particularly relevant to the Arab population in Israel and to other minority groups across the world.

1. Maintain offline communication channels and offer the option of communication by telephone (i.e., through a service call center) for populations with a low level of digital literacy and/or cultural barriers so as to ensure the accessibility and equity of telehealth services.
2. Alert health care providers to their key role in raising awareness of telehealth services and enhancing the use of the services by recommending the services to their patients (who are more likely to respond to a recommendation by a familiar health care provider).
3. Inform patients of the option of having a family member present in an online consultation with a health care provider and at the same time, clearly indicate the right to privacy in the online medical consultation (which is especially relevant to women in the Arab population).
4. Encourage health plan members to create a username and password for accessing the online services of the health plan, whether when providing routine services onsite or by referring the insured to the health plan website or application for various services.
5. Act to raise awareness of the availability of telehealth services, specifically recently introduced services such as the online ordering of medications.

## Data Availability

The dataset used and analyzed during the current study is available from the corresponding author on reasonable request.

## Footnotes

1 Muslim Women (20–29): 5.5% in study population, 12.5% in population and 13% after weights. Following the same categorical order; Muslim men (20–29): 7.5%, 13%, 13% and Druze women (20–29): 0.5%, 1.5%, 1.5%.

2 According to data of the Central Bureau of Statistics, 2019.

3 According to an Arab office holder in Israel’s health care system, Arab respondents tend to respond more openly to positively phrased survey questions.
